# Correlation between the tumoral expression of *β*3-integrin and outcome in cervical cancer patients who had undergone radiotherapy

**DOI:** 10.1038/sj.bjc.6602278

**Published:** 2004-12-14

**Authors:** G Gruber, J Hess, C Stiefel, D M Aebersold, Y Zimmer, R H Greiner, U Studer, H J Altermatt, R Hlushchuk, V Djonov

**Affiliations:** 1 Department of Radiation Oncology, University of Bern, Switzerland; 2 Pathology Laenggasse, Bern, Switzerland; 3 Institute of Anatomy, University of Bern, Baltzerstrasse 2, CH-3000 Bern 9, Switzerland

**Keywords:** *β*3-integrin expression, cervical cancer, radical radiotherapy

## Abstract

Integrins are cell-surface receptors, which mediate cell-to-cell and cell-to-extracellular matrix adhesion. Besides playing an important role in tumour angiogenesis, *β*3-integrin is also expressed in several types of epithelial cancer cells. It was the purpose of the present study to evaluate the prognostic value of *β*3-integrin expression in patients with cervical cancer. Biopsies were taken from 82 patients with squamous cell or adenocarcinomas of the uterine cervix who had undergone external-beam radiotherapy with or without brachytherapy. These tissue samples were analysed immunohistochemically for the expression of *β*3-integrin. The impact of immunoreactivity for *β*3-integrin on survival end points was assessed by univariate and multivariate analyses, and its correlation with clinicopathological characteristics evaluated by crosstabulations. *β*3-integrin was expressed in 61% (50 of 82) of the patients. Kaplan–Meier curves revealed local progression-free survival, distant metastasis-free survival and cause-specific survival to be significantly shorter (*P*-values according to the log-rank test: 0.002, 0.04 and 0.01, respectively) in patients with *β*3-integrin expression. The prognostic impact of this parameter was even higher than for other well-known prognostic parameters and remained statistically significant in the multivariate analyses. *β*3-integrin, which is expressed in the majority of patients with advanced cervical cancer, has a significant prognostic impact on outcome according to univariate and multivariate analyses.

Tumour cells are heavily influenced by their microenvironment. They derive important information not only from soluble factors, such as hormones, cytokines and growth factors, but also via direct interactions with neighbouring cells and extracellular matrix molecules.

The integrins are a family of transmembrane cell-surface receptors, which participate in cell-to-cell and cell-to-extracellular matrix interactions. They consist of *α*- and *β*-subunits. The cytoplasmic tails of the *β*-subunit contain two highly conserved motifs (NPXY and NXXY), which play a major role in the regulation of integrin-mediated functions (Dedhar and Hannigan, 1996). At least 20 different signalling proteins can associate with activated integrin receptors (Miyamoto *et al*, 1995). These cooperative interactions set in train signalling cascades, which are involved in cell differentiation, adhesion, migration, invasion, proliferation, angiogenesis and survival (for reviews, see Varner and Cheresh, 1996; Dedhar, 1999; Brakebusch *et al*, 2002). And, there is an increasing body of evidence indicating that integrins mediate the development of metastases in certain organs (Pecheur *et al*, 2002). Several of these signalling cascades involve ras or phosphatidyl inositol 3-kinase, and have been demonstrated to prevent apoptosis (for review, see Shain *et al*, 2000). Some of the aforementioned biological events are at least partially invoked by intercommunication between growth-factor receptors and integrins (Miyamoto *et al*, 1996; Eliceiri and Cheresh, 2001). There is a growing body of evidence indicating that integrins are involved in drug resistance (for a review, see Shain *et al*, 2000).

*αvβ3* is one of the most actively investigated members of the integrin receptor family. It is overexpressed in blood vessels, and together with the other known *β*-3 integrin, *αIIbβ3*, is synthesised by malignant epithelial cells. Comparative ELISA and immunoprecipitation have revealed *αvβ3* to be expressed at much higher levels in human malignant cervical tumours than in nonmalignant cervical tissue (Chattopadhyay and Chatterjee, 2001). The clinical significance has been prospectively analysed in 111 patients with melanomas of intermediate thickness. The analysis revealed the expression of *β*3-integrins to be associated with a higher risk of death (Hieken *et al*, 1996, 1999).

In the present study, we wished to ascertain whether the expression of *β*3-integrins could serve as a prognostic marker for survival in cervical cancers that had undergone radiotherapy. We also wished to determine whether the expression of *β*3-integrins is linked with that of p53, since the administration of *αvβ3* antagonists during angiogenesis is known to induce apoptosis, to selectively activate the synthesis of p53 by endothelial cells and to increase the expression of the p53-inducible cell-cycle inhibitor, p21^WAF1/CIP1^ (Stromblad *et al*, 1996).

## PATIENTS AND METHODS

In all, 91 patients who had undergone radiotherapy with a curative intent for squamous cell or adenocarcinomas of the cervix between 1990 and 1998 were eligible for this study. Individuals who had received surgical treatment for the primary tumour or the regional lymph nodes were not included. Biopsies from paraffin-embedded tissue samples were obtained with the approval of the Regional Board of the Medical Ethics Commission and with the informed written consent of the patients. In nine cases, no biopsy material was available, and these patients were excluded. Hence, 82 individuals qualified for inclusion in this study. In total, 73 of these patients had advanced FIGO stages (IIb–IVa) and nine had FIGO stages Ib–IIa. The latter nine patients were subjected to radiotherapy owing to comorbidity, which rendered their condition inoperable.

The clinical characteristics of the patient cohort are summarised in Table 1. Tumour staging was ascertained by clinical examination under anaesthesia, by chest X-radiography and by computer tomography (CT) of the pelvis, which was replaced or supplemented by MRI in several patients. Suspicious radiological findings respecting the size (>1 cm) and configuration of regional lymph nodes were not routinely substantiated by a histopathological analysis. However, borderline cases did undergo histopathological evaluation, and in seven of these positive lymph nodes were revealed. Prior to radiotherapy, all patients underwent two- or three-dimensional CT-based planning, simulator and portal-vision imaging devices being used as controls. External-beam radiotherapy (EBRT) was delivered via a 6- or 15-MV linear accelerator, a median total dose of 56 Gy (range: 31–67) being administered. A four-field box technique was used to deliver daily fractions of 1.8 or 2 Gy five times per week.

If the tumour regressed sufficiently after EBRT, then high-dose-rate brachytherapy (BT) with 192-Ir was administered. A median 192-Ir dose of 17 Gy, delivered in a median of four fractions, was given to point A. In 21 of the 82 patients, BT could not be instigated owing to the persistence of the tumoral mass and/or the destruction of the cervix, which rendered intracavitary treatment impossible. In these cases, a higher dose of EBRT was applied. The median total dose delivered to point A was 69 Gy. A total of 27 patients (33%) underwent concomitant cisplatin-based chemotherapy.

Following the course of radiotherapy, patients underwent a clinical examination and pelvic MRI or CT to define the response to treatment. The follow-up included clinical examinations in our department and/or by a gynaecologist. The mean follow-up time was 41 months (range: 3–131 months).

### Immunohistochemistry

#### *β*3-integrins:

Sections (3 *μ*m thick) of paraffin-embedded biopsy material were transferred to gelatinised microslides and air-dried overnight at 37°C. They were then dewaxed in xylene (three changes), rehydrated in ethanol and rinsed twice in Tris-buffered saline (TBS: 50 mM Tris/HCl (pH 7.4) containing 100 mM sodium chloride). Endogenous peroxidase activity was suppressed by treatment with 0.3% hydrogen peroxide (in TBS) for 10 min. Sections were then treated in a microwave oven (180 W) for 15 min and treated with 1% casein ((Sigma 8654) in TBS) for 10 min to block unspecific binding. They were then incubated with the first antibody: mouse monoclonal anti-*β*3-integrins ((*MAB2008;* Chemicon International, Temecula, CA, USA) diluted 1 : 50 in TBS) for 15 h at 4°C. Subsequently, the sections were exposed to an affinity-purified biotinylated second antibody ((anti-mouse EO 433; Dako, Glostrup, Denmark) diluted 1 : 200 in TBS) for 45 min at ambient temperature, washed three times in TBS and then treated with the avidin–biotin–horseradish peroxidase complex (P355, Dako, Glostrup, Denmark) for a similar period at the same temperature. The reaction product was visualised by exposing sections to 3-amino-9-ethylcarbazole or to 3,3-diaminobenzidine (Sigma Chemicals Company, St Louis, MI, USA). They were then mounted in Aquatex (Merck, Darmstadt, Germany). Negative controls were prepared using nonspecific mouse sera.

Initially, it was planned to evaluate the expression of *β*3-integrins within tumoral endothelial cells as well as within the epithelial ones. However, the endothelial cells seldom expressed *β*3-integrins, probably because the biopsies were usually small and therefore lacked activated capillaries characteristic for vascular ‘hot spots’ (Gasparini *et al*, 1998). Immunoreactivity for *β*3-integrins within tumoral epithelial cells was graded semiquantitatively as follows: −, undetected or negligible (<1% of the tumoral cells registering positive); +, weak (1–10% of the tumoral cells registering positive); and ++, strong (>10% of the tumour cells registering positive). When more than one biopsy per patient was available, the highest score was selected for further evaluation. The assessment was performed at a final magnification of × 200 in a blinded manner and independently by two investigators (JH, VD). Conflicting scores were resolved at a discussion microscope.

#### p53:

Sections (3 *μ*m thick) of paraffin-embedded biopsy material were treated as described for *β*3-integrins up to the end of the incubation with 0.3% hydrogen peroxide. They were then bathed in 0.01 M sodium citrate (pH 6.0), heated in a microwave oven (180 W) for 15 min and exposed to 1% casein (in TBS) for 10 min. Sections were then incubated with the first antibody: mouse anti-p53 ((DO-7. M 7001; DAKO, Glostrup, Denmark) diluted 1 : 200 in TBS) for 15 h at 4°C. Exposure to the second antibody and subsequent treatment accorded with the descriptions already given for *β*3-integrins. Negative controls were similarly prepared using nonspecific mouse sera. Sections were counterstained with haematoxylin. If more than 10% of the tumour cells exhibited intense nuclear staining, p53 was considered to have been overexpressed. When more than one biopsy per patient was available, the highest score was selected for further evaluation. Sections were assessed microscopically as described for *β*3-integrins.

### Statistics

The bivariate analysis involving the expression of *β*3-integrins and clinicopathological covariables was conducted using Fisher's exact test, the two-sided significance level being set at 5%.

For the survival analysis, three end points were used: local progression-free survival (LPFS), distant metastasis-free survival (DMFS) and cause-specific survival (CSS). Local progression during or after therapy was the determining event for LPFS, and death from the tumour (not from noncancer-related cause) for CSS. LPFS, DMFS and CSS were investigated by univariate and multivariate analyses (using the log-rank test and Cox's model, respectively). The qualifying criterion for inclusion in Cox's regression analysis was a *P*-value ⩽0.1 in the univariate analysis. A backward elimination procedure was then performed to eliminate nonsignificant parameters (*P*⩾0.1). Survival curves were plotted according to the Kaplan–Meier method, the log-rank test being used to determine significant differences between these. Statistical analyses were performed using the SPSS package (Version 11.0; SPSS Inc., Chicago, IL, USA).

## RESULTS

### Expression of *β*3-integrins

**Figure 1:**
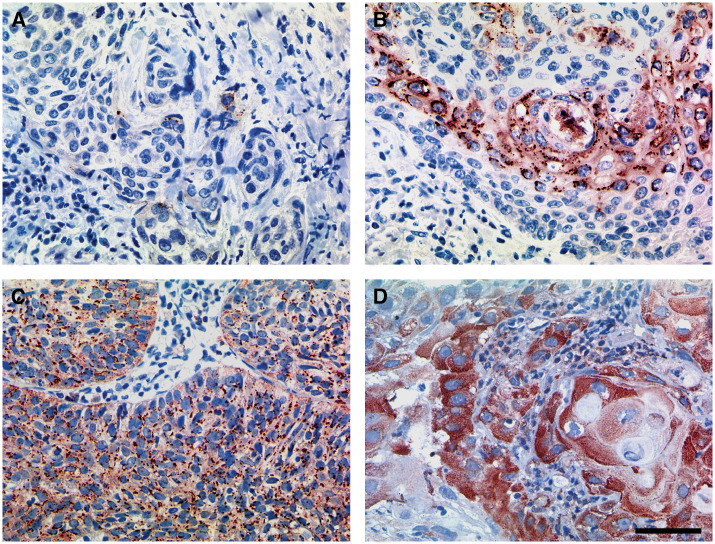
Graded semiquantitatively immunoreactivity for *β*3-integrins (brown colouration) within tumoral epithelial cells: undetected or negligible (**A**), weak (**B**) and strong (**C and D**). Bar 50 *μ*m – see (**D**).

In 32 patients (39%), *β*3-integrins were either not detected or expressed at negligible levels. In the cases that manifested clear evidence of immunoreactivity (*n*=50; 61%), 31 (38%) exhibited ‘weak’ (+) and 19 (23%) ‘strong’ (++) staining (Figure 1). Bivariate correlations revealed no significant association between the expression of *β*3-integrins and any clinicopathological parameter, including p53 expression. However, biopsies derived from patients with clinically positive lymph nodes and from those who had undergone chemotherapy were more prone to register positive for *β*3-integrins. The crosstabulations are summarised in Table 1.

### Univariate survival analysis

**Figure 2:**
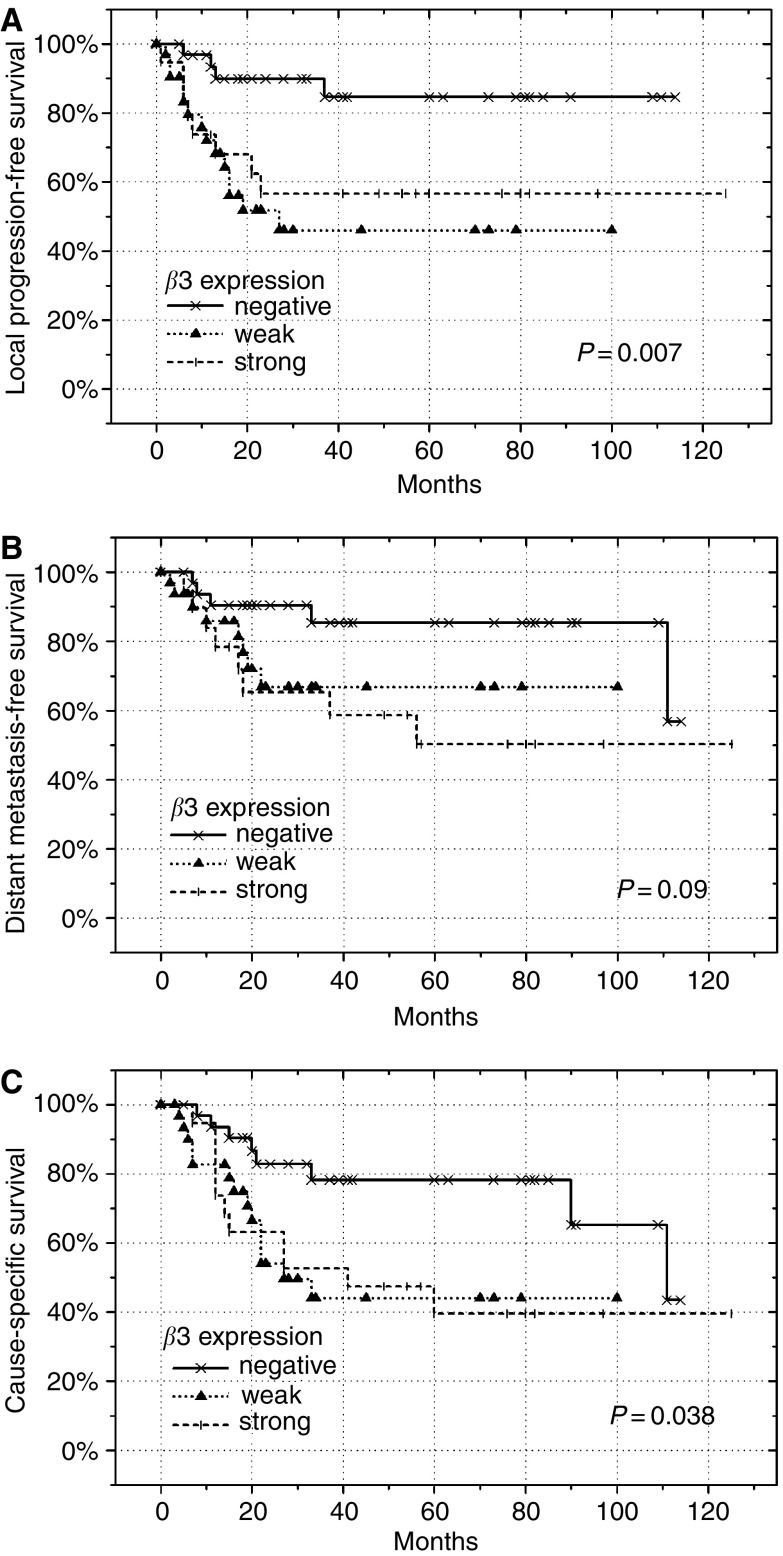
Local progression-free survival (**A**), DMFS (**B**) and CSS (**C**), expressed as a function of ‘negative’, ‘weak’ or ‘strong’ immunoreactivity for *β*3-integrins in our cohort of 82 patients with cervical cancer. *P*-values were determined according to the log-rank test.

The Kaplan–Meier survival curves (Figure 2) revealed significantly worse LPFS (*P*=0.007) and CSS (*P*=0.038), and a tendency for impaired DMFS (*P*=0.09) in patients expressing *β*3-integrins. Those with ‘weak’ or ‘strong’ *β*3-integrin expression had a similar outcome and were combined for further statistical analyses. These disclosed patients with *β*3-integrins positivity to fare significantly worse with respect to all end points (LPFS: *P*=0.002; DMFS: *P*=0.036; and CSS: *P*=0.011).

For LPFS, lymph-node status (*P*=0.009), the pretreatment level of haemoglobin (*P*=0.03) and the implementation of BT (*P*=0.009) were also significant. FIGO stage and the histological tumour type had a borderline impact (*P*=0.09 and 0.07, respectively). For DMFS and CSS, lymph-node status and histological tumour type were significant. The results of the univariate survival analysis are summarised in Table 2.

### Multivariate survival analysis

Cox's regression analysis confirmed *β*3-integrins positivity to have an independent and significant bearing on outcome (LPFS: RR=6.81, *P*=0.001; DMFS: RR=3.99, *P*=0.016; CSS: RR=4.36, *P*=0.002). The histological subtype likewise had an impact on all end-points (LPFS: RR=3.47, *P*=0.01; DMFS: RR=4.25, *P*=0.007; CSS: RR=4.52, *P*=0.001). The pretreatment level of haemoglobin was significant for LPFS (RR=0.31, *P*=0.005) and CSS (RR=0.45, *P*=0.033). Nodal status was of borderline significance for DMFS (*P*=0.06) and CSS (*P*=0.08). For LPFS, neither nodal status nor FIGO stage was important. These findings are summarised in Table 3.

## DISCUSSION

Within the large integrin family, only two of the known *α*-subunits, *αIIb* and *αv*, can dimerise with *β3*, thereby yielding *αIIbβ3* and *αvβ3*. Hitherto, *αIIbβ3* was believed to be expressed only within cells of the megakaryocyte lineage (Hynes, 1992). However, recent findings have revealed this integrin to be synthesised by a variety of tumour cells at different histological sites, such as blood, the lung, liver, kidney, colon, bladder, breast, prostate and cervix (Chen *et al*, 1997).

The integrin *αvβ3*, also known as the vitronectin receptor, has been implicated in the pathophysiology of malignant tumours. It plays an important role in angiogenesis, particularly via its expression in ‘activated’ endothelial cells (Brooks *et al*, 1994). However, the exact mechanisms underlying pro- and antiangiogenic signalling remain obscure (for a review, see Hodivala-Dilke *et al*, 2003). More recently, tumour cells have also been recognised to synthesise *αvβ3* (Chen *et al*, 1997). A possible link between its expression therein and tumour progression and/or invasiveness has been suggested for melanomas, glioblastomas and cancers of the breast, stomach and cervix (Albelda *et al*, 1990; Pignatelli *et al*, 1992; Gingras *et al*, 1995; Kawahara *et al*, 1995; Chattopadhyay and Chatterjee, 2001).

In a few studies, a significant association has been demonstrated to exist between patient survival and the expression of *αvβ3* by vascular ‘hot spots’, which served as a marker for angiogenesis (Gasparini *et al*, 1998; Vonlaufen *et al*, 2001). No data respecting a possible link between *αIIbβ3* expression and outcome have been forthcoming. In human melanomas, both the *β*3-integrins are likely to be involved in tumour progression (Trikha *et al*, 2002). In a study involving 111 patients with primary malignant melanomas, 71 (64%) were shown to express *β*3-integrins (Hieken *et al*, 1999), and these individuals were more likely to develop lung metastasis and to die of their disease (45% (32 of 71 patients)) than were those with *β*3-integrin-negative tumours (8% (three of 40 patients)), a finding that was highly significant (*P*<0.0001).

We here report for the first time that likewise in patients (most of them) with advanced stages of cervical cancer, the expression of *β*3-integrins is associated with an impaired outcome, irrespective of the level of immunoreactivity. Indeed, the significance of this factor was higher than for other prognostic parameters. Lymph-node metastasis, a very potent parameter in the univariate analysis, failed to attain significance for LPFS in Cox's regression model, which is contrary to previous findings of our group (Burri *et al*, 2003). There exist some differences in patient number and in the follow-up time, but these factors alone cannot account for the present results. The inclusion of *β*3-integrins as a new parameter in the multivariate analysis most likely diminished the importance of lymph-node status. In the present investigation, overall survival was replaced by CSS, since several patients had a high comorbidity.

Integrin positivity was related to both local and distant failure and might be more an indicator for a bad prognosis in general than a predictor for radiation response. Although a direct action of specific integrin inhibitors on tumour growth is foreseeable, a combined approach, involving integrin blockage and cytotoxic therapy, might also have a sensitising effect. In conjunction with radiotherapy, the most likely benefit to be derived from treatment with integrin inhibitors is an antiangiogenic effect. Indeed, administration of an *αvβ3* antibody/antagonist has been shown to result in the apoptosis of angiogenic but not of quiescent vascular cells (Brooks *et al*, 1994). And, function-blocking antibodies against *αvβ3* have been demonstrated to inhibit the VEGF-stimulated phosphorylation of VEGFR-2 and activation of the regulatory subunit of phosphatidyl isonitol 3-kinase (Soldi *et al*, 1999).

Several endogenous angiogenesis inhibitors may partially exert their antiproliferative effects via *αvβ3*. This has been demonstrated for endostatin (Rehn *et al*, 2001), angiostatin (Tarui *et al*, 2001), thrombospondin-1 (for a review, see Bornstein, 2001) and tumstatin (Maeshima *et al*, 2000). Indeed, when molecules such as angiostatin (Gorski *et al*, 1998; Mauceri *et al*, 1998), endostatin (Hanna *et al*, 2000) or neutralising anti-human VEGF_165_ antibodies (Gorski *et al*, 1999; Lee *et al*, 2000) are administered together with ionising radiation, the cytotoxic effects of the latter upon endothelial cells are potentiated *in vivo*, resulting in an enhanced antitumoral effect. Likewise, when radiotherapy is combined with the administration either of antibodies against VEGFR-2 or of the antiangiogenic compound TNP-470, the growth-retarding effects of radiation is potentiated (Teicher *et al*, 1996; Lund *et al*, 2000; Hess *et al*, 2001; Kozin *et al*, 2001).

As far as we are aware, only one *in vitro* study has demonstrated a synergic, retarding effect of radiotherapy and *αvβ3* blockage on tumour cell growth (Abdollahi *et al*, 2003).

And as yet, there is no evidence indicating that a blockage of *β*3-integrin expression results in radiosensitisation. Since integrin-mediated cell signalling is implicated in cell migration, invasion and survival, targeting this event could be as important as the direct inhibition of angiogenesis.

In conclusion, there was a significant relationship between *β*3-integrins expression and survival/local control in our heterogeneous group of patients with cervical cancer treated by radiotherapy. It seems worth investigating further the prognostic impact in a larger cohort of patients as it might have long-term implications in selecting patients who will do badly with standard treatment and might benefit from integrin modulation therapy.

## Figures and Tables

**Table 1:** Bivariate correlations (Fisher's exact test) between *β*3-integrin expression and patient-, tumour- and therapy-related parameters

	***β*3 expression**			
**Parameter**	**Total (%)**	**Negative (%)**	**Positive^a^ (%)**	***P*-value**
Total	82 (100)	32 (39)	50 (61)	
*Median age (yrs)*				
<63	41 (50)	13 (32)	28 (68)	
⩾63	41 (50)	19 (46)	22 (54)	0.26
				
*FIGO stage*				
I/II	42 (51)	18 (43)	24 (57)	
III/IV	40 (49)	14 (35)	26 (65)	0.50
				
*Differentiation* b				
Grade 1	3 (4)			
Grade 2	40 (55)	16 (40)	24 (60)	
Grade 3	30 (41)	12 (40)	18 (60)	1.00
				
*Lymph-node metastasis*				
Yes	32 (39)	9 (28)	23 (72)	
No	50 (61)	23 (46)	27 (54)	0.16
				
*Histology*				
Adeno/-squamous	15 (18)	7 (47)	8 (53)	
Squamous	67 (82)	25 (37)	42 (63)	0.57
				
*P53 expression* b				
Negative	54 (67)	21 (39)	33 (61)	
Positive	26 (33)	10 (38)	16 (62)	1.00
				
*Haemoglobin* b				
<Median	40 (49)	17 (42)	23 (58)	
>Median	41 (51)	14 (34)	27 (66)	0.50
				
*Median total dose (Gy)*				
⩽69	42 (51)	16 (38)	26 (62)	
>69	40 (49)	16 (40)	24 (60)	1.00
				
*Radiotherapy*				
BT	1 (1)			
EBRT+BT	60 (73)	25 (42)	35 (58)	
EBRT	21 (26)	6 (29)	15 (71)	0.43
				
*Chemotherapy*				
Yes	27 (33)	7 (26)	20 (74)	
No	55 (67)	25 (45)	30 (55)	0.10

BT=brachytherapy; EBRT=external-beam radiotherapy.

a Combined group of ‘weak’ and ‘strong’ *β*3 expression.

b Parameter was not available for all patients.

**Table 2:** Estimates of LPFS, DMFS and CSS 5 years after treatment, determined according to the Kaplan–Meier method^*^ and the univariate analyses (log-rank test^**^) for various clinicopathological characteristics in our cohort of 82 patients with cervical cancer

	5 yrs – LPFS*		5 yrs – DMFS*		5 yrs – CSS*	
**Parameter**	**%**	*P*-value**	**%**	*P*-value**	**%**	*P*-value**
*Age*		0.53		0.60		0.80
<Median	60±8		65±9		51±9	
>Median	69±8		74±8		62±8	
						
*FIGO stage*		0.09^a^		0.80		0.25
I/II	72±8		72±8		64±8	
III/IV	54±9		64±10		46±9	
						
*Histology*		0.07^a^		0.012^a^		0.016^a^
Adeno/-squamous	48±14		41±15		34±13	
Squamous	68±6		75±6		60±7	
						
*Differentiation*		0.14		0.62		0.22
Grade 2	56±9		64±10		48±9	
Grade 3	78±8		74±9		65±9	
						
*Nodal status*		0.009^a^		0.004^a^		0.002^a^
Negative	76±6		82±7		69±7	
Positive	43±10		47±11		32±10	
						
*Haemoglobin*		0.03^a^		0.89		0.09^a^
<Median	51±10		66±11		45±10	
>Median	73±7		69±8		61±8	
						
*Chemotherapy*		0.95		0.56		0.88
Yes	65±10		65±10		55±10	
No	64±7		73±7		56±8	
						
*Brachytherapy*		0.009^a^		0.98		0.16
Yes	69±6		68±7		58±7	
No	45±13		72±13		45±13	
						
*Total dose*		0.47		0.81		0.43
<Median	61±9		71±9		55±9	
>Median	67±8		67±8		57±9	
						
*P53 expression*		0.20		0.75		0.20
Negative	71±6		69±7		58±8	
Positive	51±12		71±10		51±11	
						
*β3 expression*		0.007		0.09		0.038
Negative	85±7		85±7		78±8	
Weak positive	46±10		67±10		44±10	
Strong positive	57±12		50±13		39±12	
						
*β3 expression*		0.002^a^		0.036^a^		0.011^a^
Negative	85±7		85±7		78±8	
Positive	51±8		57±9		41±8	

LPFS=local progression-free survival; DMFS=distant metastasis-free survival; CSS=cause-specific survival.

a Marked parameters were included in multivariate analysis.

**Table 3:** Multivariate analysis for LPFS, DMFS and CSS according to Cox's regression model in our cohort of 82 patients with cervical cancer

	**LPFS**	**DMFS**	**CSS**
	***P*-value**	***P*-value**	***P*-value**
	**RR (95% CI)**	**RR (95% CI)**	**RR (95% CI)**
*FIGO stage*	0.39	—	—
III/IV *vs* I/II	1.47 (0.61–3.57)		
			
*Histology*	0.01	0.007	0.001
Adeno/-squamous *vs* squamous	3.47 (1.31–9.17)	4.25 (1.50–12.04)	4.52 (1.83–11.18)
			
*Nodal status*	0.49	0.06	0.08
Positive *vs* negative	1.34 (0.59–3.03)	2.32 (0.94–5.76)	1.87 (0.92–3.81)
			
*Haemoglobin*	0.005		0.033
>Median *vs* <median	0.31 (0.14–0.71)		0.45 (0.22–0.94)
			
*Brachytherapy*	0.09	—	—
Yes *vs* no	0.49 (0.21–1.12)		
			
*β3 expression*	0.001	0.016	0.002
Positive *vs* negative	6.81 (2.21–21.00)	3.99 (1.29–12.35)	4.36 (1.73–11.00)

LPFS=local progression-free survival; DMFS=distant metastasis-free survival; CSS=cause-specific survival; RR=risk ratio; CI=confidence interval.
